# Supplementary material for: Vitality of Neural Networks under Reoccurring Catastrophic Failures
Source: Sci Rep. 2016 Aug 17;6:31674. doi: 10.1038/srep31674 (PMC4987694; doi:10.1038/srep31674)
Supplement: Supplementary Information [file srep31674-s1.pdf]

Supplementary: Vitality of Neural Networks under Reoccurring Catastrophic Failures

Shira Sardi†, Amir Goldental†, Hamutal Amir, Roni Vardi and Ido Kanter*

†These authors contributed equally to this work.

*ido.kanter@biu.ac.il

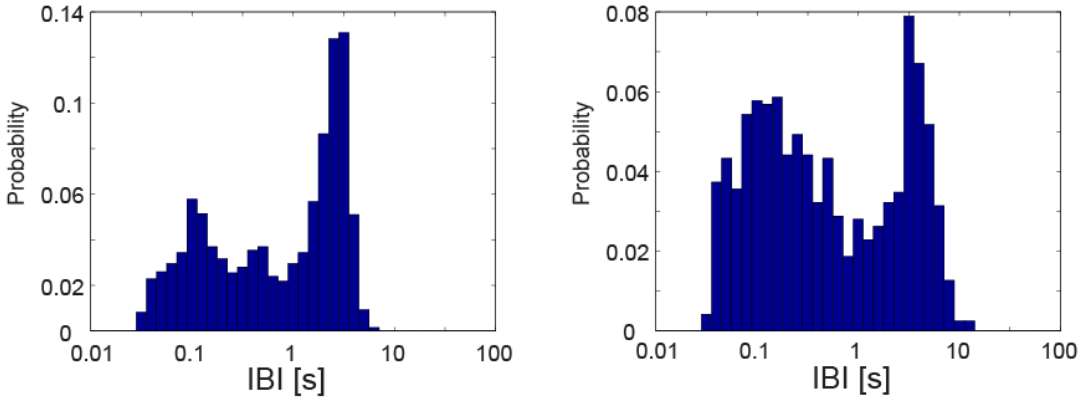

Figure S1. Inter burst intervals (IBIs) in non-blocked neural cultures. Two examples of normalized histograms for the IBIs, presented in log-scale (Online Methods), from two different non-blocked neural cultures consisting of excitatory and inhibitory connections (synapses). Results indicate two main maxima in each histogram, around 100 ms and several seconds, however, the bimodal distribution is not as clear as in excitatory networks (Fig. 1).

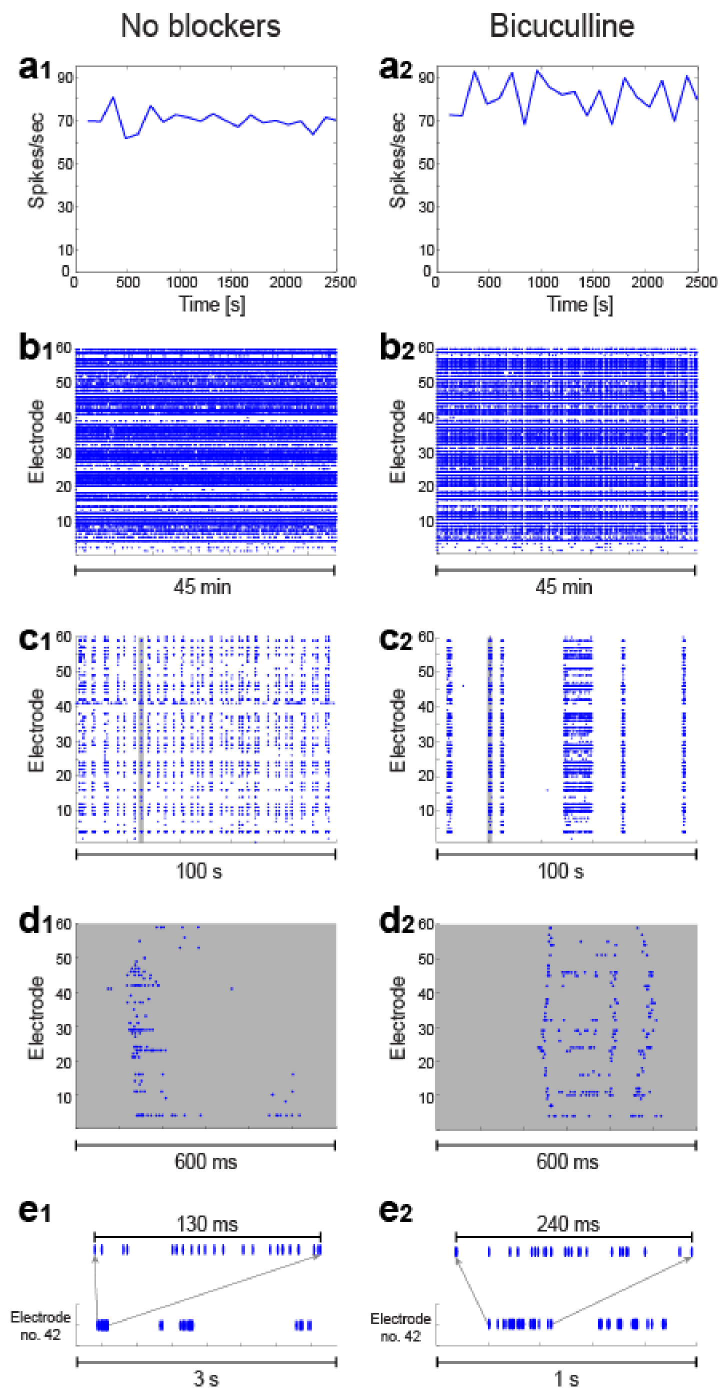

Figure S2. Spontaneous activity in neural cultures. Averaged firing rate using sliding window of 2 minutes (Online Methods) for a non-blocked culture **(a1)** and a blocked culture (where Bicuculline was added to block inhibition, Online Methods) **(a2)**. Results indicate that blocking inhibition does not substantially change the level of averaged firing activity, i.e. the average firing rate per electrode is around 1 Hz . A raster plot of the spontaneous activity, recorded from the 60 electrodes over 45 minutes, for a non-blocked culture **(b1)** and a blocked culture **(b2)**. 100 seconds out of 45 minutes in **(b1)** and **(b2)** is presented in **(c1)** and **(c2),** respectively, and a zoom in of 600 ms of the gray area **(d1)** and **(d2)**. Zoom in of one electrode recording in a non-blocked culture **(e1)** and a blocked culture **(e2)**, indicating a few dozens of recorded spikes in a burst.

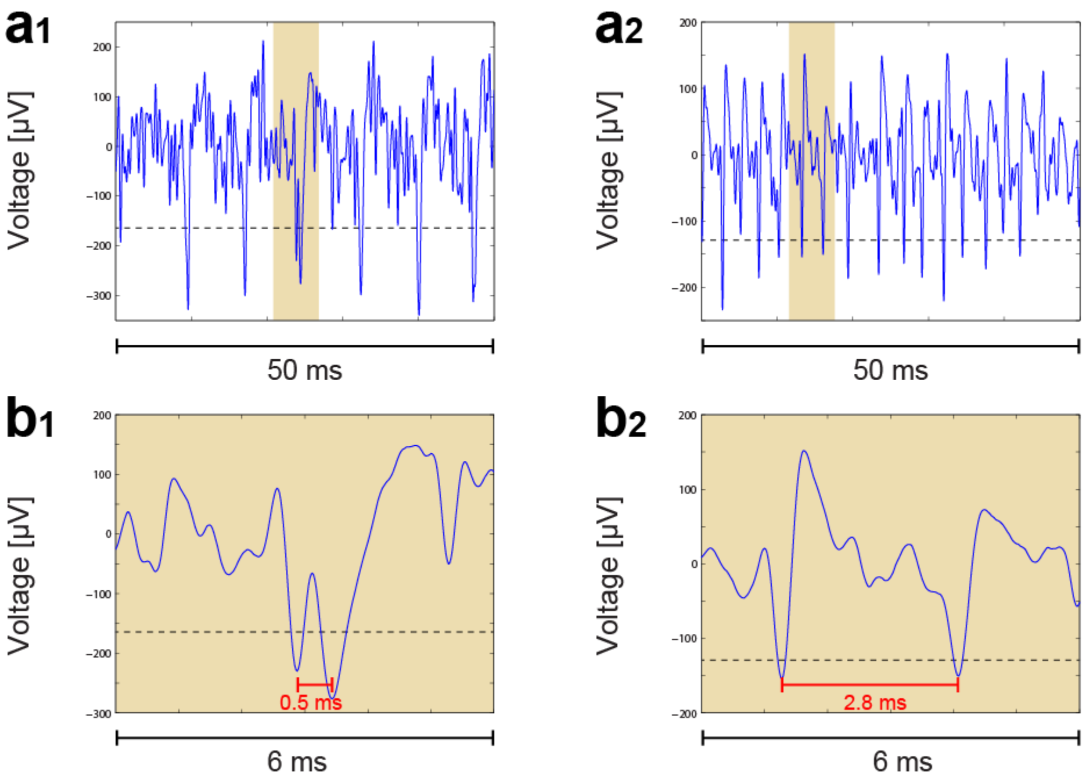

Figure S3. Voltage recordings. Recorded voltage of one electrode in a blocked culture (a cocktail of synaptic blockers was added to block excitation and inhibition, Online Methods). The black dashed line presents the threshold for spike detection (Online Methods). An electrode recording from more than one neuron **(a1)** and an electrode recording from a single neuron **(a2)**. Panels **(b1)** and **(b2)** present a zoom-in of the colored areas in **(a1)** and **(a2)**, respectively.

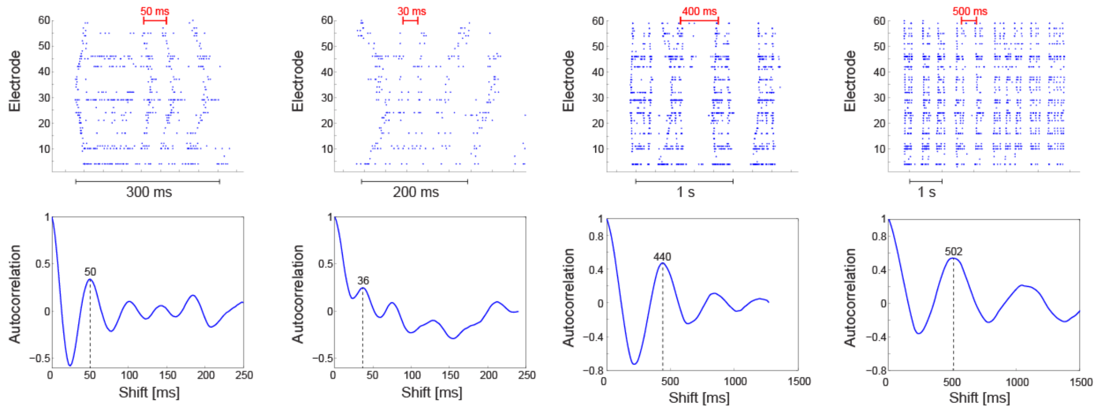

Figure S4. Autocorrelation of bursts in neural cultures. Different sets of bursts (taken from the same neural culture) separated only by short IBIs (upper panel) and the autocorrelation on their rate, respectively (lower panel). The visibility of the peaks in the autocorrelation is enhanced in comparison to Fig. 1d in the manuscript.
